# The unstable evolutionary position of *Korarchaeota* and its relationship with other TACK and Asgard archaea

**DOI:** 10.1002/mlf2.12020

**Published:** 2022-06-01

**Authors:** Yang Liu, Meng Li

**Affiliations:** ^1^ Archaeal Biology Center, Institute for Advanced Study Shenzhen University Shenzhen China; ^2^ Shenzhen Key Laboratory of Marine Microbiome Engineering, Institute for Advanced Study Shenzhen University Shenzhen China

## Abstract

The applications of marker gene concatenation have been advanced to resolve the key questions in the Tree of Life. However, the interphylum evolutionary relationship between *Korarchaeota* of TACK (*Thaumarchaeota*, *Aigarchaeota*, *Crenarchaeota*, *Korarchaeota*) and Asgard archaea remains uncertain. We applied a marker gene ranking procedure to examine their evolutionary history. Our updated trees showed confident placements of (1) *Korarchaeota* as the basal branch to other TACK archaea and as a sister group to Asgard archaea; (2) *Njordarchaeota* at basal branch to *Korarchaeota* instead of within Asgard archaea. They highlight the importance of evaluating marker genes for phylogeny inference of the Archaea domain.

The interdomain evolutionary relationships seem to be resolved by several careful standards for a robust phylogenetic reconstruction^1^, ^2^, for example, (1) inclusion of more conserved core genes with an increasing number of phylogenetically informative sites under the context of many novel lineages, (2) exclusion of genes that have undergone horizontal gene transfer (HGT), and (3) improved/balanced taxon sampling^1^. However, interphylum evolutionary relationships among *Korarchaeota* with other TACK (*Thaumarchaeota*, *Aigarchaeota*, *Crenarchaeota*, *Korarchaeota*) and Asgard archaea remain uncertain. The genomic reconstruction of the first member of the *Korarchaeota* was *Korarchaeum cryptofilum* obtained from a hot spring located in Yellowstone National Park reported in 2008; since then very few Korarchaeal genomes have been reported. According to the recent results of the 16S rRNA gene and concatenated marker genes phylogenetic analyses, *K. cryptofilum* was either (a) basal to the TACK‐Asgard groups^3^, ^4^, or (b) fell within Asgard archaea group^4^, or (c) fell within/not basal to TACK group^4^ or (d) fell between TACK and Asgard archaea^4^, ^5^, ^6^, ^7^ (Figure 1A−D). None of the above studies mentioned the unstable evolutionary position of *Korarchaeota* on the Tree of Life. Nevertheless, some phylogenetic signals also supported the nesting of the eukaryotes within the TACK as the neighbors of *Korarchaeota* ^8^, ^9^, ^10^. Subsequently, the role of *Korarchaeota* on the archaeal and even eukaryotic evolution is underestimated.

**Figure 1:**
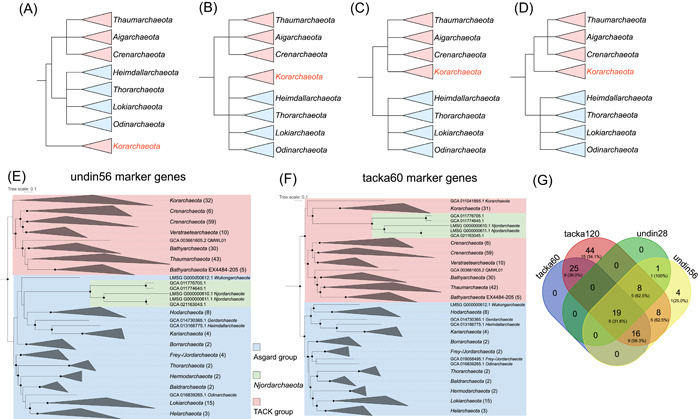
Phylogenomic illustrations, analyses, and different marker‐set comparisons. (A−D) Illustration of different scenarios of the placement of *Korarchaeota* with TACK (pink clades) and Asgard archaea groups (light blue clades). (E, F) Phylogenomic tree of TACK and Asgard archaea generated using undin56 marker gene set from Dombrowski et al.^3^ (E), and tacka60 marker gene set from this study (F). Clade colors are arranged as TACK (pink clades) and Asgard archaea groups (light blue clades). Black solid dots represent that the branch split was supported by the criteria UFBoot ≥ 90 and SH‐aLRT ≥ 90. Numbers noted after the taxonomic names indicate the numbers of genomes contained in the collapsed clades. The alignments for phylogenomic inference of undin56 and tacka60 marker gene sets contained 13,077 and 15,688 columns present in at least 60% of the taxa. (G) Venn diagram depicting the overlap among the marker gene sets of tacka60, tacka120, undin28, and undin56. Numbers in large font indicate the total number of overlapping markers. Numbers in smaller font indicate the quantity of overlapping markers that have an evolutionary history of *Njordarchaeota* neighbored with Asgard archaea, as well as their percentages to the total numbers of overlapping markers. TACK, *Thaumarchaeota*, *Aigarchaeota*, *Crenarchaeota*, *Korarchaeota*.

In this study, we sampled a large number of the most updated representative archaeal genomes from public databases, and tried to include an expanded sampling of TACK and Asgard archaea (see Supporting Information: Materials and Methods and Table S1). These genomes were then used for a preliminary phylogenomic analysis with 122 conserved single‐copy genes^11^. Most leaves of the decorated tree were in solid taxonomic agreement with the latest phylogenomic studies associated with TACK and Asgard archaeal groups^9^, ^11^ (Figure S1). However, this 122‐archaeal‐marker‐gene tree showed that the placement of *Njordarchaeota* (MAGs: GB136 and GB154), one of the recently proposed Asgard archaeal lineages^12^, was surprisingly within *Korarchaeota* clade with >90 local support values with the Shimodaira−Hasegawa test. We then ran the GTDB‐tk with “classify_wf” option to classify these genomes with the most updated reference genomes (GTDB r202, April 23, 2021). The analysis showed an incongruent classification of the two Njordarchaeal genomes, that is, GB136 as *Korarchaeia* and GB154 as *Heimdallarchaeia* (GTDB taxonomy). It is also surprising that GTDB‐tk provided dissimilar results by two independent implemented methods, that is, de_novo_wf to *de novo* infer tree on input genomes only, and classify_wf to classify input genomes by placing them on the reference tree, possibly implying a strong need to filter the marker genes when dealing with the deep‐branching archaeal lineages to increase the phylogenomic resolution.

In many cases, the phylogenetic analyses based on the friendly software PhyloSift and GTDB‐tk^13^, ^14^ would be sufficient to describe the taxonomy. However, the monophyly and the deep‐branching position of novel lineages might not be well supported^4^, ^15^. By updating the taxon sampling and inferred single‐protein trees for each marker to evaluate phylogenetic congruence and detect contaminant sequences and HGTs, Dombrowski et al.^4^ recently ranked marker genes to the extent to which they supported the monophyly of well‐established archaeal lineages. They proposed a list of the top‐ranked 25% (*N* = 28, undin28) and 50% (*N* = 56, undin56) most congruent markers, respectively, to resolve the possible positions of the archaeal root when novel DPANN (an acronym of the names of the first included phyla *Diapherotrites*, *Parvarchaeota*, *Aenigmarchaeota*, *Nanohaloarchaeota*, and *Nanoarchaeota*) archaeal group *Undinarchaeota* was included^4^.

We harnessed the two marker‐protein sets, for example, undin28 and undin56, and used the site‐heterogeneous mixture model (C60 + PMSF, posterior mean site frequency model), expecting to infer a robust placement of *Njordarchaeota*. Besides, to remove the potential deleterious effect of the oversampling of some taxa relative to others on tree topology, we subsequently down‐sampled the over‐represented phyla (e.g., *Lokiarchaeota*, *Thorarchaeota*) of the previously mentioned 952‐genome set to a 211‐genome set based on the phylogenomic tree inferred in Figure S1 (see Supporting Information: Materials and Methods). Our maximum‐likelihood inference on the undin28 marker gene set robustly placed two Njordarchaeal genomes within *Korarchaeota* with high support values 99.8/100 (UFBoot/SH‐aLRT) (Figure S2). However, the inference on the undin56 marker gene set disagreed and placed the *Njordarchaeota* as a sister group of *Wukongarchaeota*, which is another recently proposed Asgard archaeal phylum^9^ (Figure 1E). Nevertheless, the support values of the undin56 tree were 43/70 (UFBoot/SH‐aLRT), which are below the bottom‐line of the typical criteria (UFBoot ≥ 95 and SH‐aLRT ≥ 80) to trust a clade^16^. The topology of the undin56 marker gene tree (Figure1E) seems to be akin to the trees inferred by Xie et al.^12^ in terms of the position of the *Njordarchaeota*. Notably, the study by Xie et al. did not include any *Korarchaeota* genomes; thus, the placement of *Njordarchaeota* might still be mysterious.

Not only *Njordarchaeota* but also *Korarchaeota* has faced a similar situation. As shown in Figure 1A−D, we have observed in various literature that the position of *Korarchaeota* was differently placed in terms of their relatives to other archaea in TACK and Asgard superphyla. Considering these abovementioned incongruent phylogenomic analyses, we performed an in‐depth analysis to filter out the top‐ranked taxon‐specific marker proteins for TACK and Asgard archaeal groups, to remove the phylogenetic signals that contribute to topological incongruencies (see Supporting Information: Materials and Methods).

We firstly collected a pool of 248 nonredundant marker proteins from seven previous archaeal/universal phylogenomic studies^4^, ^6^, ^9^, ^10^, ^13^, ^17^, ^18^ (Table S2). Notably, among the 248 nonredundant marker proteins, 12 asCOGs (Asgard archaeal Cluster of Orthologous Groups) were included, which were derived from 209 asCOGs for Asgard archaeal phylogeny generated by Liu et al.^9^, to better resolve the relationship between *Njordarchaeota* and other Asgard archaea. We finally generated two marker gene sets, that is, tacka60 (top 25% ranked marker genes, *N* = 60, including three asCOGs) and tacka120 (top 50% ranked marker genes, *N* = 120, including six asCOGs). We added extra Korarchaeotal genomes that are not representative species in GTDB for undin56 and tacka60 marker gene sets, and increased the size of outgroup genomes to 36 genomes that closely related to TACK as outgroups to alleviate a possible long‐branch attraction effect by distant outgroups^19^. The maximum‐likelihood inferences of the phylogeny based on both the tacka60 and tacka120 marker gene sets showed a confident placement of *Korarchaeota* as the basal branch to other TACK archaea and as a sister group to Asgard archaea as depicted in Figure 1D but with additional Asgard archaeal lineages. Such evolution position of *Korarchaeota* is following several recent studies about archaeal/universal Tree of Life^4^, ^5^, ^6^, ^7^. Considering the most ancient lineage within TACK archaea so far, *Korarchaeota* might play a transitional role in the evolution and diversification between TACK and Asgard archaea.

Besides, these phylogenomic analyses all showed a congruent result: placing *Njordarchaeota* within *Korarchaeota* with high support values (tacka60: 97/98, tacka120: 100/94, UFBoot/SH‐aLRT) (Figures 1F and S3). We subsequently examined the tendency of each marker protein toward the position of *Njordarchaeota* as relative to either *Korarchaeota* or Asgard archaea. As shown in Figure 1G, we compared the four newly ranked marker sets, that is, undin28, undin56, tacka60, and tacka120. We found that they shared a core set of 19 marker proteins as 31.6% of them designate *Njordarchaeota* with Asgard archaea. The additional marker proteins introduced by undin28 and undin56 had a higher portion (56.3%−62.5%) of the proteins that favor cluster *Njordarchaeota* with Asgard archaea, whereas tacka60 and tacka120 only introduced 34.1%−36.0% (as the same level as the core 19 marker proteins) of the extra marker proteins with the same tendency. It seems that the marker sets tacka60 and tacka120 are unbiased with the position of *Njordarchaeota*. These results seem to resolve the position of *Njordarchaeota*. However, they cannot explain why only the two Njordarchaeotal genomes but no other Korarchaeotal species wandered around the tree of TACK and Asgard archaeal groups. On the one hand, without additional Njordarchaeotal genomes, we did not think that our analyses here could robustly fix the position of *Njordarchaeota*, which is one of the potentially underrepresented novel phyla. On the other hand, the analysis of eukaryotic‐signature proteins (ESPs) on the Njordarchaeotal genomes seems to support the original notion proposed by Xie et al.^12^ that *Njordarchaeota* is indeed one of the Asgard archaeal phylum.

The presence of ESPs in TACK and Asgard archaeal genomes is a common feature particularly shared by these two archaeal superphyla^20^. However, Asgard archaea accumulated a significant number of ESPs compared to the primordial ones in TACK archaea. The endosomal sorting complex required for transport (ESCRT) machinery has been thought to be eukaryotic specific but found in TACK and Asgard archaea^9^, ^18^, ^21^, ^22^. ESCRT‐III, as one of the main subcomplexes of ESCRT machinery, is a central player in ESCRT function that mediates remodeling and scission of endomembrane^23^. The cell division (Cdv) systems in Asgard archaea have several homologs of eukaryotic ESCRT‐III subunits (CdvBs), whereas those in TACK archaea harbor archaeal CdvB^21^. These previous studies supported the notion that only Asgard archaea possess eukaryotic ESCRT‐III subunits.

Therefore, we performed a search for proteins containing Snf7 domain (CdvB) in the 952 genome set to have the most up‐to‐date understanding of CdvB distribution among TACK and Asgard archaea. The investigation did not find any CdvB homologs in *Korarchaeota* genomes. Meanwhile, it obtained a CdvB homolog encoded in one of the *Njordarchaeota* genomes (GB154). A further phylogenetic analysis inferred that this CdvB homolog belonging to one of the CdvB classes Vps20/32/60 in *Njordarchaeota* was clustered with Asgard archaea and seems to be closely related to the eukaryotic ones (Figure S4). This result supports the claim made by Xie et al.^12^ that *Njordarchaeota* is one of the Asgard archaeal lineages. In addition, we investigated the distribution of ESPs in *Korarchaeota*, *Njordarchaeota* and six Asgard archaeal genomes sequenced from isolates reported from two recent studies^24^, ^25^. The UPGMA dendrogram of the ESP distribution showed that all the five Njordarchaeotal genomes were not clustered with the Asgard archaeal isolates (Figure S5). However, we cannot rule out the possibility that these ESPs in *Njordarchaeota* had been horizontally transferred from other Asgard archaea, highlighting *Njordarchaeota* might play a transitional role in the evolution and diversification between *Korarchaeota* and Asgard archaea.

In summary, we sampled a large number of the most updated archaeal genomes from public databases, and tried to include an expanded sampling of TACK and Asgard archaea. We collected 11 sets of gene markers broadly used in literature and pooled them into a nonredundant 248 gene marker set. We applied a marker gene ranking procedure to examine the evolutionary history of the 248 gene marker set. We identified the top 25% (tacka60) and top 50% (tacka120) ranked markers that satisfied reciprocal monophyly based on the extent to which they supported the well‐established archaeal relationships within TACK and Asgard archaea. Finally, we used these markers to estimate an updated phylogenetic tree of TACK‐Asgard archaea. The updated tree showed a confident placement of *Korarchaeota* as the basal branch to other TACK archaea and as a sister group to Asgard archaea. Besides, it robustly placed a newly proposed “*Njordarchaeota*” within *Korarchaeota*. However, one of the *Njordarchaeota* genomes encoded a Snf7 domain protein (Vps20/32/60) that is a close relative to Asgard archaea. Additionally, *Njordarchaeota* seems to have a distribution of ESPs encoded in their genomes similar to Asgard archaea, highlighting *Njordarchaeota* might play a transitional role in the evolution and diversification between *Korarchaeota* and Asgard archaea. Therefore, their underestimated potential transition role in the evolutionary history of TACK and Asgard archaea deserves further investigation. Finally, we propose to include additional marker genes to resolve a more accurate evolutionary position of deep‐branching *Korarchaeota* and point out the necessity to re‐rank the marker genes to deal with unresolved basal lineages.

## AUTHOR CONTRIBUTIONS

Yang Liu designed the research, performed the analyses, and wrote the paper. Meng Li provided guidance and wrote the paper.

## ETHICS STATEMENT

Not applicable.

## CONFLICT OF INTERESTS

The authors declare no conflict of interests.

## Supporting information

Supporting information.

Supporting information.

Supporting information.

Supporting information.

Supporting information.

Supporting information.

Supporting information.

## Data Availability

All the data including sequence alignments, trees, and hmm profiles used for marker identifications have been deposited in the FigShare repository at 10.6084/m9.figshare.17126846.
