# Supplementary material for: The unstable evolutionary position of Korarchaeota and its relationship with other TACK and Asgard archaea
Source: mLife. 2022 Jun 1;1(2):218–22. doi: 10.1002/mlf2.12020 (PMC10989867; doi:10.1002/mlf2.12020)
Supplement: Supplementary file 1 — Supporting information. [file MLF2-1-218-s003.pdf]

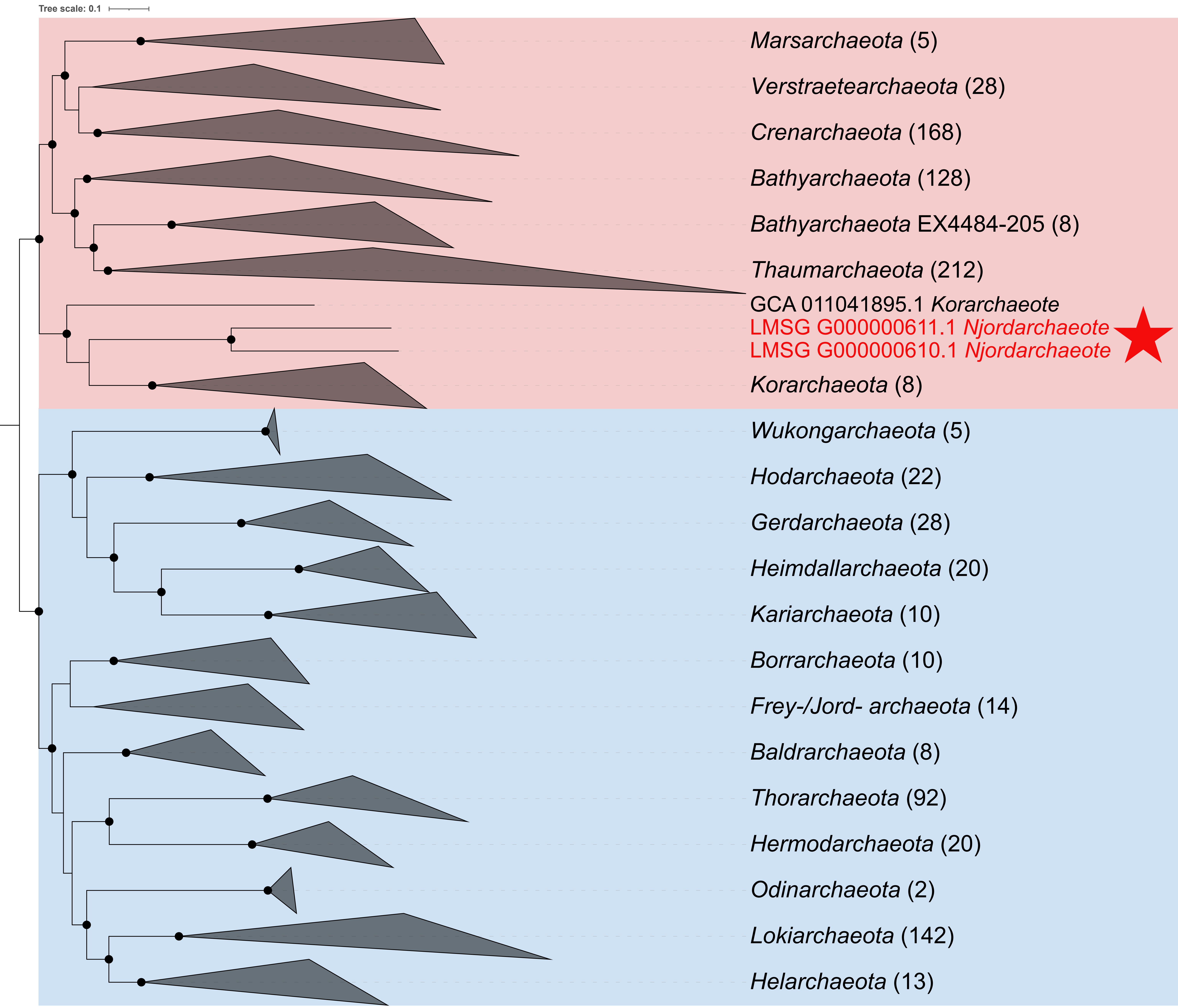

Supplementary Figure 1 Phylogenomic tree generated using 122 archaeal marker genes (GTDB-tk). Clade colors are arranged as TACK (pink clades) and Asgard archaea groups (light blue clades). Black solid dots represented the branch split was supported by the criteria SH-aLRT >= 90. Numbers noted after the taxonomic names indicated the numbers of genomes containing in the collapsed clades. The alignment for phylogenomic inference contained 5124 columns present in at least 60% of the taxa.
