# Supplementary material for: The unstable evolutionary position of Korarchaeota and its relationship with other TACK and Asgard archaea
Source: mLife. 2022 Jun 1;1(2):218–22. doi: 10.1002/mlf2.12020 (PMC10989867; doi:10.1002/mlf2.12020)
Supplement: Supplementary file 3 — Supporting information. [file MLF2-1-218-s005.pdf]

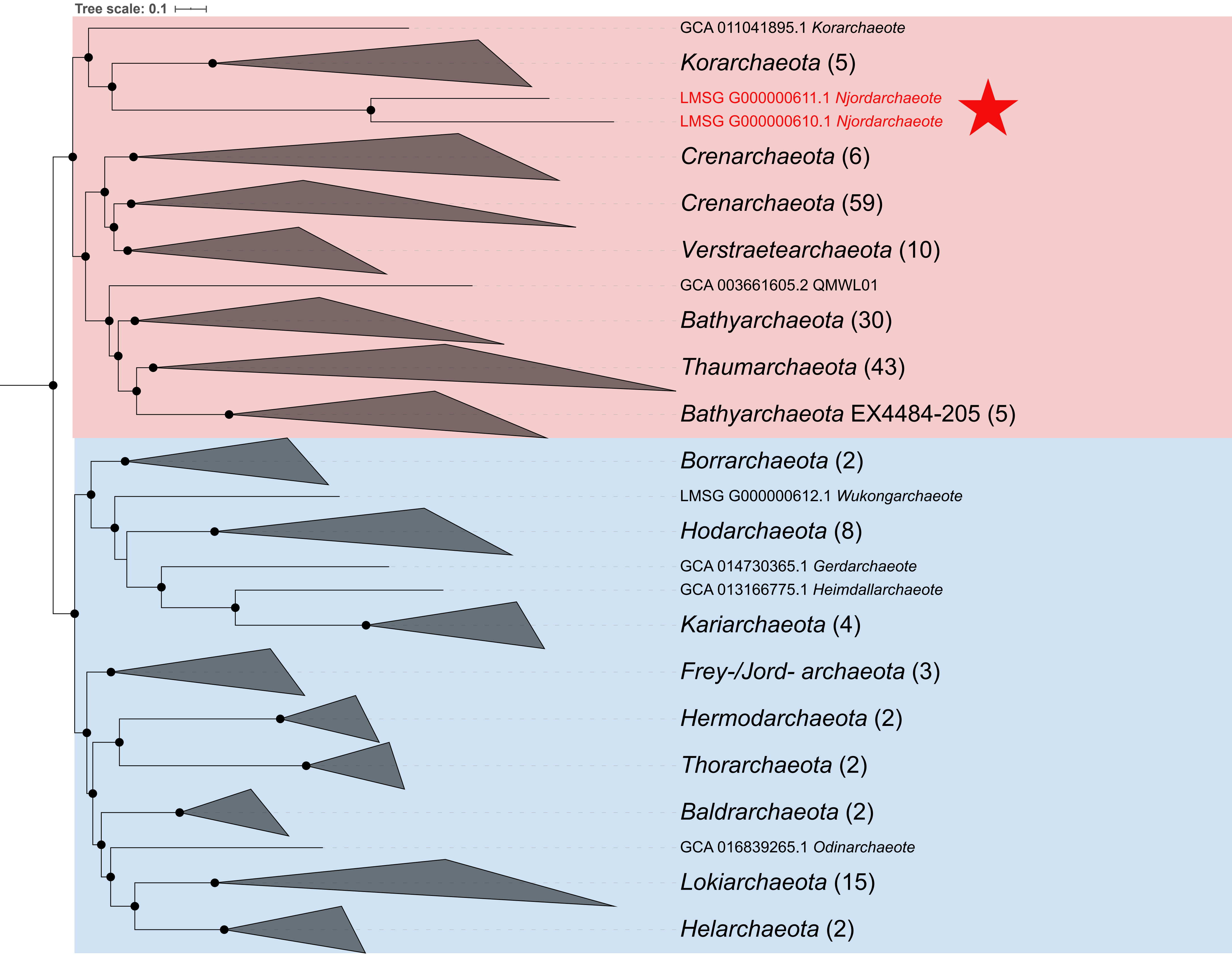

Supplementary Figure 3 Phylogenomic tree of TACK and Asgard archaea generated using tacka120 marker gene set from this study. Clade colors are arranged as TACK (pink clades) and Asgard archaea groups (light blue clades). Black solid dots represented the branch split was supported by the criteria UFBoot >= 90 and SH-aLRT >= 90. Numbers noted after the taxonomic names indicated the numbers of genomes containing in the collapsed clades. The alignments for phylogenomic inference of tacka120 marker gene sets contained 29,557 columns present in at least 60% of the taxa.
