# Supplementary material for: The unstable evolutionary position of Korarchaeota and its relationship with other TACK and Asgard archaea
Source: mLife. 2022 Jun 1;1(2):218–22. doi: 10.1002/mlf2.12020 (PMC10989867; doi:10.1002/mlf2.12020)
Supplement: Supplementary file 4 — Supporting information. [file MLF2-1-218-s004.pdf]

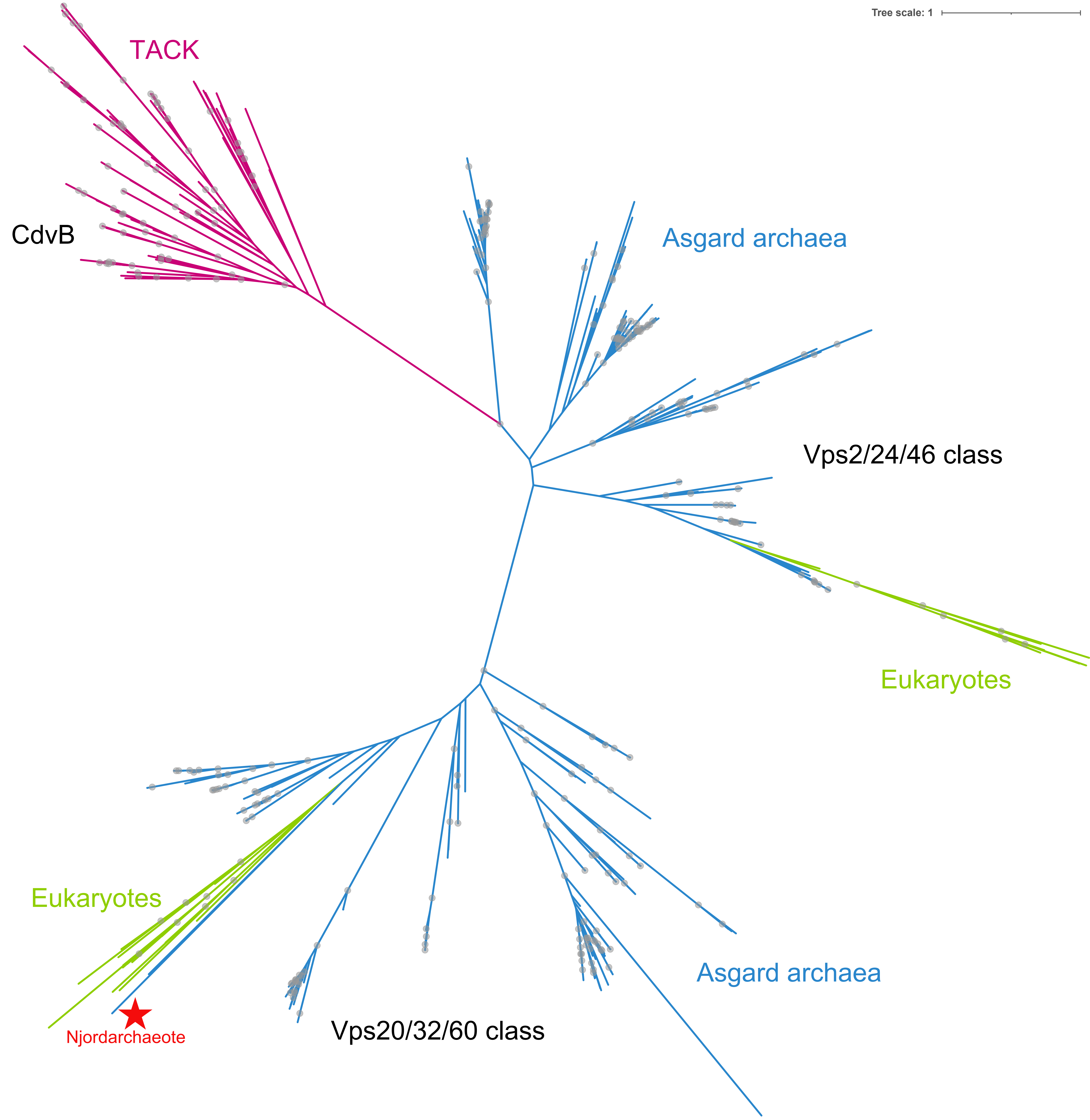

Supplementary Figure 4 Phylogenetic tree of Snf7 domain proteins in TACK and Asgard archaea. Grey solid dots represented the branch split was supported by the criteria UFBoot >= 90 and SH-aL-RT >= 90. The alignments for phylogenetic inference Snf7 domain protein sequences contained 134 columns.
