# Supplementary material for: The unstable evolutionary position of Korarchaeota and its relationship with other TACK and Asgard archaea
Source: mLife. 2022 Jun 1;1(2):218–22. doi: 10.1002/mlf2.12020 (PMC10989867; doi:10.1002/mlf2.12020)
Supplement: Supplementary file 7 — Supporting information. [file MLF2-1-218-s002.docx]

**Materials and Methods**

**Genome data**

We firstly retrieved 559 genomes that are classified as the species representatives in the phylum Thermoproteota (GTDB taxonomy, TACK superphylum in NCBI taxonomy), a recent reclassification that united the TACK superphylum (Rinke *et al.*, 2021). We obtained 388 Asgard archaeal genomes from NCBI Genbank and eLMSG databases (Xie *et al.*, 2021). Subsequently, we pooled these genomes into a 952 genome-set, including five as outgroup: Halobacteriota, Thermoplasmatota, Hydrothermarchaeota, and Hadarchaeota and Methanobacteriota (GTDB taxonomy, Halobacteria, Thermoplasmata, Hydrothermarchaea, Hadesarchaea and Methanobacteria in NCBI taxonomy) (Table S1). Additionally, 36 genomes closely related to TACK (Stygia: *N* = 5, Acherontia: *N* = 12, Methanomada: *N* = 19, Table S1) were used as outgroups in Figure 1E and 1F. Treemmer v0.3 was used to reduce the size of the 952 genome-set with minimal loss of diversity to a 211-genome set with option “-RTL 0.6” (Menardo *et al.*, 2018).

**COG assignment for marker genes**

Amino acid sequences of the proteins used for phylogenetic analyses were predicted by Prodigal (v2.6.3) (Hyatt *et al.*, 2010) embedded in GTDB-tk (v1.7.0) (Chaumeil *et al.*, 2019). The hmm profiles of arCOGs and TIGRFAMs were obtained from eggNOG 5.0 (Huerta-Cepas *et al.*, 2019) and NCBI RefSeq(Haft *et al.*, 2013; Li *et al.*, 2020), respectively. The Asgard asCOGs hmm profiles were generated by profiling the original alignments from Liu et al. using hmmer v3.3.2 (Eddy, 1998; Liu *et al.*, 2021). Sequences were assigned a COG/protein family by using hmmsearch v3.3.2 and filtered out the sequences with > 1E-5 sequence E-value and > 1E-4 domain E-value. The sequences passed the criteria were used for subsequent phylogenetic analyses.

**Ranking markers based on split score**

Each of the 248 marker gene sequences was aligned using MAFFT-linsi (Katoh and Standley, 2013) and poorly-aligned positions were removed with BMGE “-m BLOSUM30 -h 0.55” (Criscuolo and Gribaldo, 2010). We inferred initial maximum likelihood trees for all 248 marker genes using IQ-Tree v2.1.4 “-m LG+G4+F -bb 1000 -wbtl -bnni” (Nguyen *et al.*, 2015), to inspect whether a single gene tree failed to meet reciprocal phylum-level monophyly. We applied a marker gene ranking procedure (Dombrowski *et al.*, 2020; Moody *et al.*, 2021). It calculated the split score to rank each of the 248 marker genes that satisfied reciprocal monophyly based on the extent to which they recovered established phylum-level relationships within TACK and Asgard archaea (Table S3). The scripts quantify the number of splits, or occurrences where a taxon fails to gather within its expected taxonomic lineage, across all gene phylogenies. We ranked the 248 marker genes using the following split-score criteria: the number of splits per taxon and the splits normalized to the species count. The percentage of split phylogenetic groups was used to determine the highest ranking (top 25% and top 50%) marker genes.

**Phylogenetic analyses**

The trimmed 122 archaeal marker-gene concatenation of 5124 columns was obtained by implementing the GTDB-tk “denovo_wf” methods. The phylogenomic tree of the 122 archaeal marker-gene concatenation was inferred with FastTree2 with “-wag -gamma -spr 4 -pseudo -mlacc 2 -slownni” (Price *et al.*, 2010). Phylogenomic trees of undin28, undin56, tacka60 and tacka120 marker gene sets were inferred with IQ-Tree by employing C60 mixture model and PMSF approximation invoked with a starting tree “-ft” inferred by FastTree2 as describe above. The Snf7 domain protein sequences (PF03357.24) was extracted by filtering the hmmsearch results with “-cut_ga” as validation cut-off. The aligning and trimming jobs followed the above methods. The phylogenetic tree of Snf7 domain proteins was inferred by using IQ-Tree under LG+G4+F model.

**Plotting**

Statistical analyses were performed using R version 3.6.3 (Team, 2020). Trees were visualized with Interactive Tree of Life v5 (Letunic and Bork, 2021).

**References**

1. Chaumeil, P.-A., Mussig, A.J., Hugenholtz, P., and Parks, D.H. (2019) GTDB-Tk: a toolkit to classify genomes with the Genome Taxonomy Database. *Bioinformatics* 36: 1925–1927.
2. Criscuolo, A. and Gribaldo, S. (2010) BMGE (Block Mapping and Gathering with Entropy): a new software for selection of phylogenetic informative regions from multiple sequence alignments. *BMC Evol Biol* 10: 210.
3. Dombrowski, N., Williams, T.A., Sun, J., Woodcroft, B.J., Lee, J.-H., Minh, B.Q., et al. (2020) Undinarchaeota illuminate DPANN phylogeny and the impact of gene transfer on archaeal evolution. *Nat Commun* 11: 3939.
4. Eddy, S.R. (1998) Profile hidden Markov models. *Bioinform Oxf Engl* 14: 755–63.
5. Haft, D.H., Selengut, J.D., Richter, R.A., Harkins, D., Basu, M.K., and Beck, E. (2013) TIGRFAMs and Genome Properties in 2013. *Nucleic Acids Res* 41: D387–D395.
6. Huerta-Cepas, J., Szklarczyk, D., Heller, D., Hernández-Plaza, A., Forslund, S.K., Cook, H., et al. (2019) eggNOG 5.0: a hierarchical, functionally and phylogenetically annotated orthology resource based on 5090 organisms and 2502 viruses. *Nucleic Acids Res* 47: D309–D314.
7. Hyatt, D., Chen, G.-L., LoCascio, P.F., Land, M.L., Larimer, F.W., and Hauser, L.J. (2010) Prodigal: prokaryotic gene recognition and translation initiation site identification. *BMC Bioinformatics* 11: 119.
8. Katoh, K. and Standley, D.M. (2013) MAFFT Multiple Sequence Alignment Software Version 7: Improvements in Performance and Usability. *Mol Biol Evol* 30: 772–780.
9. Letunic, I. and Bork, P. (2021) Interactive Tree Of Life (iTOL) v5: an online tool for phylogenetic tree display and annotation. *Nucleic Acids Res* 49: W293–W296.
10. Li, W., O’Neill, K.R., Haft, D.H., DiCuccio, M., Chetvernin, V., Badretdin, A., et al. (2020) RefSeq: expanding the Prokaryotic Genome Annotation Pipeline reach with protein family model curation. *Nucleic Acids Res* 49: gkaa1105-.
11. Liu, Y., Makarova, K.S., Huang, W.-C., Wolf, Y.I., Nikolskaya, A.N., Zhang, X., et al. (2021) Expanded diversity of Asgard archaea and their relationships with eukaryotes. *Nature* 593: 553–557.
12. Menardo, F., Loiseau, C., Brites, D., Coscolla, M., Gygli, S.M., Rutaihwa, L.K., et al. (2018) Treemmer: a tool to reduce large phylogenetic datasets with minimal loss of diversity. *BMC Bioinformatics* 19: 164.
13. Moody, E.R.R., Mahendrarajah, T.A., Dombrowski, N., Clark, J.W., Petitjean, C., Offre, P., et al. (2021) An estimate of the deepest branches of the tree of life from ancient vertically-evolving genes. *Biorxiv* 2021.01.19.427276.
14. Nguyen, L.-T., Schmidt, H.A., Haeseler, A. von, and Minh, B.Q. (2015) IQ-TREE: A Fast and Effective Stochastic Algorithm for Estimating Maximum-Likelihood Phylogenies. *Mol Biol Evol* 32: 268–274.
15. Price, M.N., Dehal, P.S., and Arkin, A.P. (2010) FastTree 2 – Approximately Maximum-Likelihood Trees for Large Alignments. *PLOS One* 5: e9490.
16. Rinke, C., Chuvochina, M., Mussig, A.J., Chaumeil, P.-A., Davín, A.A., Waite, D.W., et al. (2021) A standardized archaeal taxonomy for the Genome Taxonomy Database. *Nat Microbiol* 6: 946–959.
17. Team, R.C. (2020) R: A Language and Environment for Statistical Computing.
18. Xie, R., Wang, Y., Huang, D., Hou, J., Li, L., Hu, H., et al. (2021) Expanding Asgard members in the domain of Archaea sheds new light on the origin of eukaryotes. *Sci China Life Sci* 1–12.
