# Supplementary material for: The unstable evolutionary position of Korarchaeota and its relationship with other TACK and Asgard archaea
Source: mLife. 2022 Jun 1;1(2):218–22. doi: 10.1002/mlf2.12020 (PMC10989867; doi:10.1002/mlf2.12020)
Supplement: Supplementary file 5 — Supporting information. [file MLF2-1-218-s001.pdf]

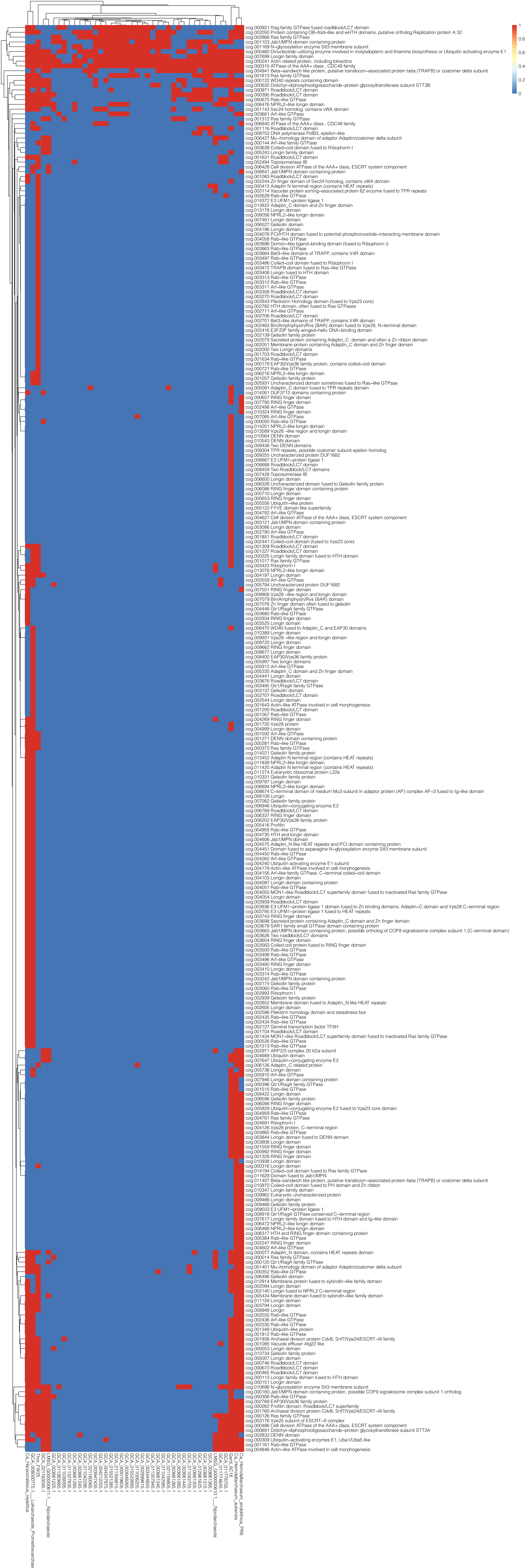

Supplementary Figure 5 Heatmap of distribution of Eukaryotic signatured proteins among 34 Korarchaeal genomes and two Njordarchaeal genomes as well as six Asgard archaeal genomes sequenced from isolates cultured from two studies (Imachi et al 2020 PMID: 31942073, Wu et al 2022 PMID: 35027677). Their proteomes were searched against the ESP database generated by Liu et al. (PMID: 33911286) by hmmsearch with evalue < 1E-20 as cutoff. The hierarchical trees were generated using UPGMA (unweighted pair group method with arithmetic mean) methods.
